# The Diagnostic Value of Trace Metal Concentrations in Hair in Carotid Artery Disease

**DOI:** 10.3390/jcm12216794

**Published:** 2023-10-27

**Authors:** Anna Olasińska-Wiśniewska, Tomasz Urbanowicz, Anetta Hanć, Jolanta Tomczak, Beata Begier-Krasińska, Andrzej Tykarski, Krzysztof J. Filipiak, Patrycja Rzesoś, Marek Jemielity, Zbigniew Krasiński

**Affiliations:** 1 Department of Cardiac Surgery and Transplantology, Poznan University of Medical Sciences, 61-848 Poznan, Poland; turbanowicz@ump.edu.pl (T.U.);; 2 Department of Trace Analysis, Faculty of Chemistry, Adam Mickiewicz University, 61-614 Poznan, Poland; 3 Department of Vascular and Endovascular Surgery, Angiology and Phlebology, Poznan University of Medical Sciences, 61-848 Poznan, Poland; 4 Department of Hypertensiology, Angiology and Internal Medicine, Poznań University of Medical Sciences, 61-848 Poznan, Poland; 5 Institute of Clinical Science, Maria Sklodowska-Curie Medical Academy, 00-136 Warsaw, Poland; 6 Poznań University of Medical Sciences, 61-848 Poznan, Poland

**Keywords:** carotid artery stenosis, trace, biomarker, zinc, chrome, copper

## Abstract

Several studies showed the role of trace elements in the increase in human susceptibility to cardiovascular diseases. Carotid artery stenosis is a leading cause of ischemic neurological events. We aimed to analyze the potential role of trace elements in hair as biomarkers of atherosclerotic carotid artery disease. Materials and Methods: Fifty-seven (n = 31 (54%) men and n = 26 (46%) women) individuals with a mean age of 67.7 ± 7.7 years who were white, European, non-Hispanic, and non-Latino were diagnosed and treated in hypertensiology/internal medicine and surgical departments over three consecutive months. Of these patients, forty were diagnosed with advanced carotid artery disease, and seventeen comprised a group of healthy controls. Inflammatory and oncological diseases were exclusion criteria. Hair samples were collected, and 14 trace elements were analyzed. Clinical and laboratory data were compared and revealed differences in the co-existence of diabetes (*p* = 0.036) and smoking history (*p* = 0.041). In the multivariable analysis, zinc, chrome, and copper revealed predictive value for the occurrence of carotid artery disease, and their combined receiver operating curve showed area under the curve of 0.935, with a sensitivity of 95% and a specificity of 82.4%. Conclusion: Our report shows the significance of trace elements analyses in patients with advanced carotid artery disease. We revealed that zinc, copper, and chrome concentrations are of particular importance in differentiating atherosclerotic disease and may serve as biomarkers of carotid atherosclerosis. Hair samples represent an easily obtained and beneficial biomatrix for the assessment of biomarkers.

## 1. Introduction

The balance of trace elements in the human body is influenced by the chronic environmental exposure through water and food consumption. Essential trace metals, including zinc, iron, copper, play significant roles in in cellular catalytic, regulatory, or signaling processes [1,2]. In healthy tissues, metal homeostasis is strictly controlled. However, disturbed homeostasis may lead to derangements in physiological pathways and the development of oncological or metabolic diseases [1,3]. Decreased selenium and zinc concentrations were noted in patients with coronavirus 2019 disease (COVID-19) [4]. Importantly, toxic metals compete with essential metals [5], e.g., mimicking them by binding and interacting with the same enzymes and therefore interfering with normal reactions. Conversely, some essential metals (e.g., calcium and magnesium) may compete with toxic metals, preventing toxic-metal-induced tissue damage and serving an antioxidant function.

Several studies have evaluated the role of trace elements in the increase in human susceptibility to cardiovascular diseases [6,7,8]. While serum is the most common material in the majority of studies, hair samples have recently garnered significant interest as a source of information. The metal content in human blood may be influenced by the time of the day, meals eaten, serum protein concentration, etc. [9,10], whereas hair is independent of homeostasis and therefore appears to be more useful than blood in detecting mineral deficiencies and diseases in humans. Hair samples may present higher concentrations of metals (e.g., zinc), which are less labile and reflect recent exposure [9,11]. A typically tested sample of hair is 3–4 cm long, so analysis results show the average concentration of mineral nutrients assimilated over the past 3–4 months [12]. Therefore, the significance of hair sampling and analysis for the diagnosis of atherosclerosis is a remarkable direction of investigation [9]. Carotid artery stenosis characterized by the atherosclerotic narrowing of one or both carotid arteries is related to the occurrence of cerebral ischemic events [13]. Our aim was to analyze trace elements in hair samples obtained from patients with carotid artery stenosis and healthy controls.

## 2. Materials and Methods

Fifty-seven white, European, non-Hispanic, and non-Latino patients (n = 31 (54%) male and n = 26 (46%) female) with a mean age of 67.7 ± 7.7 years were enrolled in the analysis. They were diagnosed and treated in hypertensiology/internal medicine and surgical departments over three consecutive months (December 2022/February 2023). Advanced carotid artery disease referred for vascular intervention was a criterion for inclusion in the carotid group for 40 patients (70%) and a lack of any atherosclerotic changes determined 17 patients (30%) to be members of the control group. Inflammatory and oncological diseases were exclusion criteria. Out of the carotid group, 35 patients (87%) underwent surgical carotid atherectomy, and 5 more underwent percutaneous interventions. The procedures and postoperative recovery were uneventful.

### 2.1. Hair and Blood Samples Analysis

Hair and blood samples were collected upon admission. Patient demographic and clinical data were obtained and analyzed together with biochemical results. Blood samples were collected before the procedure under identical conditions. A simple whole blood analysis (neutrophil count, lymphocyte count, monocyte count, hemoglobin, platelets, and relevant indexes—the neutrophil-to-lymphocyte ratio (NLR), monocyte-to-lymphocyte ratio (MLR), and platelet-to-lymphocyte ratio (PLR)) was conducted and serum creatinine was immediately measured using a routine hematology analyzer (Sysmex Euro GmbH, Norderstedt, Germany). The GFR was calculated using the simplified Modification of Diet in Renal Disease (MDRD) formula.

Hair samples (approx. 0.5 g from each patient) were cut from the scalp, above the neckline, and stored in a plastic container at room temperature until analysis. The hair samples were washed sequentially via stirring with a 0.5% Triton X-100 solution, 5% acetone solution, and deionized water. The washed hair was dried and cut into smaller pieces and digested in a high-pressure closed microwave digestion system (Ethos One, Milestone, Sorisole, Italy), according to a procedure described previously [14]. The digested hair samples were analyzed using an inductively coupled plasma mass spectrometer (ICP-MS 7100x Agilent, Santa Clara, CA, USA) for the detection of the following elements: lithium (Li), natrium (Na), aluminum (Al), calcium (Ca), titanium (Ti), chrome (Cr), manganese (Mn), nickel (Ni), copper (Cu), zinc (Zn), selenium (Se), strontium (Sr), molybdenum (Mo), and cadmium (Cd). The instrumental setting and data validation were as described in [14].

### 2.2. Analytical Figures of Merit

The validity of the analytical method was assessed by analyzing the certified reference material (CRM), NCS ZC 81002b Human Hair (Beijing, China). The reference material was digested according to the same procedure as the hair samples. Validation parameters such as linearity, precision, the limit of detection (LOD), and trueness were evaluated. The linearity of the calibration curves, calculated as the correlation coefficient R, was greater than 0.9996 for all analytes. The LOD was defined as 3.3 s/b where s is the standard deviation corresponding to 10 blank injections and b is the slope of the calibration graph. The LOD values were in a range from 0.006 µg/g for Cd to 10 µg/g for Ca. Precision values were calculated as the coefficient of variation (CV) (%) and ranged from 1.5% to 3.4% for all elements. Trueness was evaluated by applying the certified reference material and expressed as recovery values (%) ranging from 94% to 107%.

### 2.3. Statistical Analysis

The normality of the distribution of the variables was tested using the Shapiro–Wilk test. The *t*-test, Cochran–Cox test, Mann–Whitney test, or Fisher’s exact test were used where applicable to compare the variables between two groups. Logistic regression was performed to analyze the laboratory data which predicted the occurrence of carotid artery disease. We reported odds ratio (ORs) with 95% confidence intervals (CIs). A receiver operator characteristic (ROC) analysis was carried out. A Spearman correlation analysis was used to describe the correlation between the variables. A statistical analysis was performed using JASP statistical software (JASP Team (2020). JASP (Version 0.13.1) (Computer software)). A *p* < 0.05 was considered statistically significant.

This study was approved by the Institutional Ethics Committee (No 875/22, dated 3 November 2022) and respected the principles outlined in the Declaration of Helsinki. All patients gave their written informed consent for hair and blood sampling and analysis.

## 3. Results

The entire study group was characterized by co-existing comorbidities, including arterial hypertension (n = 45, 79%), hypercholesterolemia (n = 45, 79%), diabetes mellitus (n = 20, 35%), chronic obstructive pulmonary disease (COPD) (n = 3, 5%), and kidney dysfunction (n = 3, 5%), defined as a glomerular filtration rate (GFR) below 60 mL/min. The carotid and control groups were matched as there were no differences regarding age (*p* = 0.150), sex (*p* = 0.566), or non-vascular diseases, including arterial hypertension (*p* = 1.000) and hypercholesterolemia (*p* = 0.146). The patients differed in terms of the co-existence of diabetes mellitus (*p* = 0.036) and smoking history (*p* = 0.041). The carotid (group 1) and control (group 2) groups did not differ significantly in terms of laboratory data (Table 1).

The patients’ medical histories regarding pharmacotherapy were collected as follows: statins (n = 30 (75%) vs. n = 13 (77%) in group 1 vs. group 2, respectively (*p* = 1.000)), angiotensin converting enzyme inhibitor (ACE-I) or angiotensin receptor blockers (ARB) (n = 25 (63%) vs. n = 10 (59%) in group 1 vs. group 2, respectively (*p* = 1.000)), calcium-channel blockers (n = 17 (43%) vs. n = 2 (12%) in group 1 vs. group 2, respectively (*p* = 0.032)), beta-blockers (n = 21 (53%) vs. n = 4 (24%) in group 1 vs. group 2, respectively (*p* = 0.078)), metformin (n = 6 (15%) vs. n = 4 (24%) in group 1 vs. group 2, respectively (*p* = 0.464)), and dapagliflozin (n = 3 (8%) vs. n = 2 (12%) in group 1 vs. group 2, respectively (*p* = 0.629)). The analysis of the concentration of trace elements in the hair and scalp was performed and compared between both groups (carotid disease vs. control), and the results are presented in Table 2.

In the univariable analysis, data which differed between groups were included (Table 3).

**Figure 1:**
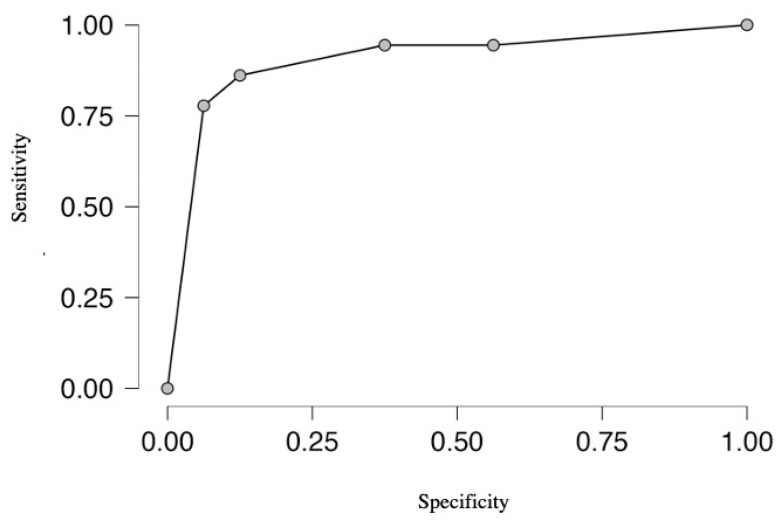
The receiver operating characteristic analysis (ROC) showed the combined value of zinc, copper, and chrome for the prediction of carotid artery stenosis.

The combined value of zinc, copper, and chrome for the prediction of carotid artery stenosis evaluated in the receiver operating characteristic analysis (ROC) showed an area under the curve (AUC) of 0.935 with 95% sensitivity and 82.4% specificity (Figure 1). The zinc concentration correlated with the chrome concentration (*p* < 0.001; Spearman’s rho 0.627)) and copper concentration (*p* < 0.001; Spearman’s rho 0.601). The copper/zinc ratio did not differ between subgroups (*p* = 0.580).

Finally, we analyzed the concentrations of trace metal elements in smokers with advanced carotid disease and non-smokers in the healthy group to assess the differences between subjects with the potentially harmful influence of external factors. We revealed statistically significant differences between smokers and healthy non-smokers in aluminum (median (Q1–Q3) 311 (184–621) mg/kg vs. 44 (32–109) mg/kg, *p* < 0.001), calcium (median (Q1–Q3) 3212 (1953–3636) mg/kg vs. 1119 (730–2245) mg/kg, *p* = 0.035), chrome (median (Q1–Q3) 8.174 (5.310–11.460) vs. 2.149 (1.538–4.434) mg/kg, *p* = 0.004) and zinc (median (Q1–Q3) 172.6 (157.3–215.3) mg/kg vs. 110.2 (10.9–135.5) mg/kg, *p* < 0.001) hair concentrations (mg/kg).

## 4. Discussion

The results of our study demonstrate that elevated hair zinc, copper, and chrome concentrations may characterize patients with carotid artery stenosis.

Atherosclerosis is related to changes in the whole cardiovascular system; however, carotid, coronary, and lower limb diseases are of particular interest due to their high prevalence rates in developed communities and high risks of complications. The more advanced changes are recognized, the more complex therapy is necessary, including non-pharmacological, pharmacological, and surgical options. Therefore, early diagnosis is important for the prompt implementation of prophylaxis and therapy. The identification of markers of a disease is beneficial and may modify the diagnostic process [15]. New evidence suggests the significance of toxic and trace elements in the development and progression of cardiovascular atherosclerosis [16]. An imbalance in a metal concentration may lead to impaired immune function and the accumulation of immune complexes [5]. Consequently, the uncontrolled release of inflammatory cytokines, renal damage, and the stimulation of nervous system may provoke the development or exaggeration of cardiovascular diseases. Hair samples may be easily obtained and processed for the determination of trace element concentrations. Several studies proposed mineral analyses of hair in correlation with oncological [17,18,19] neurological [20,21], or metabolic [2,22,23] diseases. Trace element concentrations in blood, hair, urine, or vessel samples were also analyzed in cardiovascular diseases, including coronary and carotid artery disease and aortic valve disease [8,24,25,26].

In our analysis, patients with and without carotid atherosclerosis differed in their hair concentrations of several trace elements, including aluminum, calcium, chrome, copper, and zinc. In multivariable and ROC analyses, chrome, copper, and zinc occurred as valuable predictors of carotid artery disease and therefore may be proposed as potential hair biomarkers of carotid atherosclerosis.

Zinc plays an essential role in the catalytic function and structure of over 70 enzymes, acting in the metabolism of different proteins, lipids, and carbohydrates, gene regulation, neurotransmission, and the structure of chromatin [1,10,27]. Zinc exerts antioxidant and anti-inflammatory effects [27] via the modulation of oxidation damage as zinc ions displace iron and copper from oxidation-vulnerable sites in low-density lipoprotein cholesterol particles, liposomes, and red blood cell membranes [28,29]. A decrease in the serum zinc ion concentration causes the dysfunction of the cell-mediated immune response [10] and enhances oxidative stress-related signaling processes in the endothelium. A zinc imbalance may influence the maintenance of endothelial cell integrity [30]. Zinc deficiency was associated with the occurrence of atherosclerosis and coronary artery disease [27]. Oxidative-stress-related factors are activated during the inflammatory response in the background of the development and progression of atherosclerosis. Thus, zinc, which inhibits oxidative processes, plays a protective role in the occurrence of atherosclerosis. In animal models, zinc deficiency enhanced the oxidation of low-density lipoprotein cholesterol. In contrast to such observations, other authors reported the enhancement of atherosclerosis via increased oxidant generation and decreased high-density lipoproteins [31].

In our analysis, the median zinc concentration was similar (slightly higher) to data presented by Dziedzic et al. [11]. The authors showed no difference, but similar concentrations were found in acute coronary syndrome and stable coronary syndrome subgroups, 166 (39–285) ppm vs. 166.5 (25–495) ppm, respectively (*p* = 0.937). Interestingly, in our analysis, the “healthy group” presented a lower concentration of zinc. Similarly, in a study by Białkowska et al. [32], the results of analyses of hair zinc concentration and the zinc/copper ratio (Zn/Cu) in patients with myocardial infarction were significantly higher than in controls. These observations may be partially explained by the study performed by Stadler et al. [29]. The authors presented elevated concentrations of both zinc and iron in advanced carotid artery and abdominal aorta lesions compared to early changes and healthy tissue. Moreover, a highly significant correlation was found between zinc and calcium accumulation. It was concluded that a high concentration of zinc may be an indicator of calcium accumulation, fibrosis, and a decreased propensity to lesion rupture. A high zinc concentration may promote lesion stability. Other studies [33,34] in which an LA-ICP-MS technique was used to conduct direct elemental analyses in the soft tissues of the arterial wall and calcified plaque showed that the highest Zn signals were measured in the area connecting the arterial wall and the atherosclerotic plaque. This may be due to the presence of increased amounts and activity of matrix metalloproteinases in the artery wall. In a study by Tasic et al. [25], atherosclerotic plaques obtained during carotid endarterectomy had higher concentrations of zinc if calcification dominated over the fibrolipid structure. The authors found no difference between serum zinc concentrations in atherosclerotic and normal subjects. We suppose that a high hair concentration of zinc may reflect a greater accumulation of zinc in tissues, including carotid arteries in our analysis, even though zinc deficiency may be observed in the blood.

Copper plays a crucial role in metabolism, serving as a cofactor of various enzymes, including copper/zinc superoxide dismutase, lysyl oxidase, and ceruloplasmin [9,32]. It presents, however, a dual, anti- and pro-atherogenic effect [25]. An increased concentration of copper may lead to the increased production of reactive oxidative species and promote oxidative stress [9]. The role of serum copper concentration in atherosclerotic disease was already presented in our previous study [8]. A higher copper concentration was associated with an increased inflammatory response in patients referred for surgical coronary revascularization. Baj et al. [35] determined the mineral profiles of liver and brain tissues collected at autopsy. They reported statistically significant higher concentrations of calcium and copper in the atherosclerosis group compared to patients with hepatic steatosis and concluded that the involvement of these elements is essential in the pathogenesis of atherosclerosis. In other reports, serum copper was related to an increased cardiovascular risk and higher mortality [36] and aortic aneurysm [37]. Interestingly, Radak et al. [38] showed a significantly lower copper concentration in ulcered atherosclerotic plaque in comparison with a control healthy group. The serum copper concentration was higher in patients with hemorrhagic plaque than in patients with calcified plaque [25]. Shokrzadeh et al. [39] presented a higher copper concentration in patients with ischemic cardiomyopathy. Dziedzic et al. [9] did not prove a significant association between hair copper content and risk factors for coronary artery disease. A meta-analysis by Chen et al. [40] showed no association between hair copper concentration and myocardial infarction, though the geographical subgroup sub-analysis revealed a higher copper concentration in Pakistan patients with a history of myocardial infarction. The authors suggested ethnic differences in copper concentration.

We report a lower hair copper concentration as a prognostic marker of the severity of carotid artery disease. Several reports [9,32] underline the significance of the copper/zinc ratio, pointing out requirement for a combined assessment of these trace elements in cardiovascular risk assessment. Keeping in mind the interaction between zinc and copper in oxidation-sensitive sites, a joint analysis seems reasonable. Nevertheless, in our analysis, the copper/zinc ratio did not differentiate patients with carotid stenosis and healthy controls.

The discrepancies in the reports presenting a positive, negative, or neutral role of copper in different biomatrixes may result from the dual effect of copper in the cellular metabolism. Further studies are necessary to enable an innate explanation of these phenomena. In our opinion, hair samples are valuable since they enable the evaluation of long-lasting exposure.

Chrome has multiple oxidation states [41]. The chrome III and VI states are the most common [42]. Chrome III serves as a component or activator of enzymes in redox reactions in the cell [43] and in carbohydrate and lipid metabolism [5]. It is involved in the maintenance of glucose metabolism as a second messenger in response to insulin in the cell [44]. Chrome VI is related to several diseases, including dermatitis, respiratory disorders, cancer, and kidney disease, and it is treated as carcinogen [42,45,46]. Briefly, mechanisms of chrome VI toxicity include deoxyribonucleic acid (DNA) damage, genomic instability, and the generation of reactive oxygen species [41]. Urine and blood chrome samples reflect relatively short-term exposure [42], and biomonitoring the different states of chrome is complicated. Due to their slow growth rate, toenails are believed to present evidence of long-term exposure [42], and hair samples may most likely serve as a similarly good biological matrix. A lower serum chrome concentration was independently associated with the presence of coronary artery disease in Alissa et al.’s [47] analysis of 130 Saudi men with a history of myocardial infarction compared to 130 age-matched healthy controls. According to Guallar et al. [48] the toenail chrome concentration is inversely associated with the risk of myocardial infarction in men. In this study, participants with hypertension had lower chrome concentrations than those without such a history.

Our results suggest that a higher hair chrome concentration is associated with carotid artery disease. This observation remains in contrast to previous studies involving serum, urine, and toenail chrome concentrations.

Additionally, since we noticed smoking as a parameter which differed in both subgroups, though it did not represent a predictive factor in the multivariable analysis, we evaluated differences in trace metal concentrations between the two most opposite subpopulations—healthy non-smokers and smokers with advanced carotid disease. This subanalysis revealed higher concentrations of aluminum, calcium, zinc, and chrome in the diseased group. Unkiewicz-Winiarczyk et al. [49] reported higher concentrations of heavy metals, including aluminum, cadmium, and lead, in the hair of tobacco smokers and lower concentrations of bioelements (calcium, magnesium, iron, zinc, and copper), which was explained by a decrease in suppleness due to a lower appetite and reduced absorption due to disturbances in the digestive system [50]. Skalny et al. [51] showed an association between smoking and the hair chrome concentration and inverse association with hair iron and selenium concentrations. The authors underlined the possible mediation of the health hazards of smoking via an alteration in the essential metal metabolism. The occurrence of atherosclerotic disease was not present in these studies. Thus, based on our results, we suggest that the profile of trace metal elements is profoundly related to inflammatory and mineral changes in the body’s homeostasis and to the development of carotid artery disease.

We present the diagnostic value of trace metal elements in the hair of patients with or without advanced carotid artery disease. Currently, the progress in imaging diagnostics of atherosclerotic diseases is substantial and includes different imaging modalities, such as ultrasonography, computed tomography, and magnetic resonance. Nevertheless, screening protocols require easy and reliable methods for the early differentiation of subjects at a higher risk of disease occurrence. Most prophylactic programs use simple markers to indicate the population at risk and there is a requirement for further, more advanced diagnostics or treatment. Kadogou et al. [52] presented the benefits of the cardio–ankle vascular index (CAVI) and biochemical biomarkers (matrix metalloproteinases) for the assessment of the severity of atherosclerosis and the prediction of major adverse cardiovascular events after carotid revascularization, describing “vulnerable patients”. Our previous report [53] proved the significant value of lower counts of large unstained cells obtained via an analysis of the morphology of blood cells for differentiating patients with a higher probability of carotid artery occlusion.

We believe that further development of hair metal analysis and its implementation in clinical daily practice would be beneficial in the management of carotid artery disease. Hair samples, which are easily available and may be stored without specific requirements, are a particularly valuable source of biomarkers. Currently, the availability of centers which can perform hair metal elements analyses is low; thus, the further implementation of hair samples in diagnostics should be postulated.

The lack of serum concentrations of trace metal elements to confirm our hypothesis is a limitation of our study but still appears to be worthy of further analysis. The other limitation is the lack of a possibility of evaluating the histological structure of atherosclerotic plaques in our patients.

## 5. Conclusions

Our report shows the significance of trace elements analyses in patients with advanced carotid artery disease. We revealed that zinc and chrome concentrations are of particular importance in differentiating atherosclerotic disease and may serve as biomarkers of carotid atherosclerosis. Hair samples are easily obtained and can provide useful pre-diagnostic information, serving as a beneficial biomatrix for the assessment of biomarkers and assisting in the prediction of the disease risk.

## Figures and Tables

**Table 1:** Clinical and laboratory characteristics of patients presenting with carotid disease (group 1) and the control group (group 2).

Parameter	Group 1 Carotid Group n = 40	Group 2 Control Group n = 17	*p*
Age (years) (median (Q1–Q3))	68 (64.5–73)	65.5 (59.8–69.3)	0.150
Sex (male (%)/female (%))	23 (58)/17 (42)	8 (47)/9 (53)	0.566
Comorbidities:			
Arterial hypertension, n (%)	32 (80)	14 (82)	1.000
Hypercholesterolemia, n (%)	30 (75)	16 (94)	0.146
Diabetes mellitus, n (%)	11 (28)	10 (59)	0.036 *
COPD, n (%)	1 (3)	2 (12)	0.209
Active smoking, n (%)	11 (28)	5 (30)	1.000
Smoking (ever), n (%)	25 (63)	5 (30)	0.041 *
Laboratory results—median (Q1–Q3))			
White blood cells (×10^9^/L)	8.45 (6.83–9.70)	8.18 (6.26–9.46)	0.598
Neutrophil count (×10^9^/L)	5.29 (4.29–6.85)	4.6 (3.95–6.45)	0.509
Lymphocyte (×10^9^/L)	2.09 (1.63–2.64)	1.82 (1.35–2.40)	0.235
Monocyte (×10^9^/L)	0.43 (0.36–0.58)	0.54 (0.42–0.66)	0.125
NLR	2.32 (1.88–3.46)	2.29 (1.87–3.58)	0.879
MLR	0.20 (0.18–0.30)	0.30 (0.20–0.47)	0.090
PLR	119 (100–167)	127 (106–173)	0.496
Hemoglobin (mmol/L)	8.7 (8.1–9.3)	8.7 (8.3–9.1)	0.768
Hematocrit (%)	41 (−9–44)	43 (41–48)	0.076
Platelet count (×10^9^/L)	248 (199–298)	222 (206–255)	0.340
MPV (fl)	8.0 (7.8–8.7)	8.7 (8.4–9.4)	0.057
RDW (%)	13.6 (13.3–14.4)	13.8 (13.4–14.3)	0.583
Serum lipid profile—median (Q1–Q3))			
Total cholesterol (mmol/L)	3.7 (3.1–4.0)	4.11 (3.7–4.3)	0.800
HDL (mmol/L)	1.1 (1–0–1.4)	1.0 (0.9–1.5)	0.842
LDL (mmol/L)	2.2 (1.4–2.6)	2.3 (2.0–2.7)	0.364
TG (mmol/L)	1.2 (1.0–1.6)	1.2 (1.0–1.4)	0.934
Serum creatinine (umol/L) (median (Q1–Q3))	83 (72–97)	97 (76–116)	0.149

Abbreviations: COPD–chronic obstructive pulmonary disease; HDL—high-density lipoprotein cholesterol; LDL—low-density lipoprotein cholesterol; MLR—monocyte/lymphocyte ratio; MPV—mean platelet volume; NLR—neutrophil/lymphocyte ratio; PLR—platelet/lymphocyte ratio; TG—triglycerides; RDW—red blood cells distribution width. * Statistically significant.

**Table 2:** Concentrations of trace elements in the hair (mg/kg) in patients presenting with carotid disease (group 1) and a control group (group 2).

Parameter	Group 1 N = 40	Group 2 N = 17	*p*
Trace elements:			
Li	0.092 (0.057–0.178)	0.084 (0.052–0.114)	0.345
Na	1020 (659–1373)	761 (731–793)	0.083
Al	351 (182–594)	40 (18–108)	<0.001 *
Ca	3197 (1847–4420)	1026 (422–2076)	0.001 *
Ti	6.492 (4.59–10.174)	7.306 (3.211–8.328)	0.421
Cr	8.269 (5.202–12.052)	1.852 (1.286–3.752)	<0.001 *
Mn	1.136 (0.652–1.838)	1.143 (0.621–1.879)	0.621
Ni	4.168 (2.419–7.152)	3.351 (2.173–4.012)	0.158
Cu	50.739 (33.174–78.328)	25.273 (19.265–55.679)	<0.001 *
Zn	181.3 (155.1–217.9)	110.2 (10.9–135.5)	<0.001 *
Se	0.511 (0.212–1.355)	0.484 (0.334–0.640)	0.547
Sr	5.500 (2.552–13.469)	8.398 (4.345–9.629)	0.067
Mo	0.532 (0.286–0.737)	0.288 (0.120–0.457)	0.078
Cd	0.025 (0.017–0.038)	0.031 (0.013–0.062)	0.547

* Statistically significant.

**Table 3:** Univariable and multivariable analyses.

	Univariable			Multivariable		
	OR	95% CI	*p*	OR	95% CI	*p*
Clinical:						
Diabetes	0.266	0.081–0.872	0.029 *	-	-	-
Smoking (ever)	4.00	1.176–13.603	0.026 *	-	-	-
Trace elements						
Al	1.007	1.002–1.012	0.005 *	-	-	-
Ca	1.000	1.000–1.001	0.093	-	-	-
Cr	1.299	1.079–1.565	0.006 *	1.832	1.194–2.810	0.006 *
Cu	1.009	0.994–1.024	0.246	0.943	0.905–0.983	0.006 *
Zn	1.024	1.010–1.038	0.001 *	1.027	1.010–1.044	0.001 *

Abbreviations: CI–confidence interval. OR—Odds ratio. * Statistically significant.

## Data Availability

The data presented in this study are available upon request from the corresponding author.
